# Health-Related Quality of Life and Dietary Supplement Use in Physically Active People and Athletes: A Cross-Sectional Study

**DOI:** 10.3390/sports13090321

**Published:** 2025-09-11

**Authors:** Walter Sapuppo, Davide Giacconi, Antonietta Monda, Antonietta Messina, Daniele Saccenti, Claudia Maria Mineo, Maria Casillo, Salvatore Allocca, Giovanni Michelini, Regina Gregori Grgič, Vincenzo Monda, Jacopo Lamanna, Mattia Ferro, Girolamo Di Maio, Marcellino Monda, Marco La Marra

**Affiliations:** 1 Department of Psychology, Sigmund Freud University Wien, Ripa di Porta Ticinese 77, 20143 Milan, Italy; w.sapuppo@milano-sfu.it (W.S.); giacconi.ext@milano-sfu.it (D.G.); saccenti.phd@milano-sfu.it (D.S.); mineo.ext@milano-sfu.it (C.M.M.); g.michelini@milano-sfu.it (G.M.); r.gregori@milano-sfu.it (R.G.G.); m.ferro@milano-sfu.it (M.F.); 2 Studi Cognitivi, Cognitive Psychotherapy School and Research Center, Foro Buonaparte 57, 20121 Milan, Italy; 3 INSPIRE Lab, Department of Psychology, Sigmund Freud University Wien, Ripa di Porta Ticinese 77, 20143 Milan, Italy; 4 Department of Human Science and Quality of Life Promotion, San Raffaele Telematic University, 00166 Rome, Italy; antonietta.monda@uniroma5.it; 5 Department of Precision Medicine, University of Campania “Luigi Vanvitelli”, 80138 Naples, Italy; antonietta.messina@unicampania.it; 6 Department of Experimental Medicine, University of Campania “Luigi Vanvitelli”, 80138 Naples, Italy; maria.casillo@unicampania.it (M.C.); salvatore.allocca@unicampania.it (S.A.); marcellino.monda@unicampania.it (M.M.); 7 Department of Disabilities, Fondazione Istituto Ospedaliero di Sospiro, 26048 Sospiro, Italy; 8 Department of Economics, Law, Cybersecurity, and Sports Sciences, University of Naples “Parthenope”, 80035 Nola, Italy; vincenzo.monda@uniparthenope.it; 9 Faculty of Psychology, Vita-Salute San Raffaele University, 20132 Milan, Italy; lamanna.jacopo@hsr.it; 10 Department of Psychology and Health Sciences, Pegaso Telematic University, 80143 Naples, Italy

**Keywords:** dietary supplements, physical activity, health-related quality of life (HRQoL), athletes, supplement use behavior

## Abstract

The use of dietary supplements is widespread among athletes and physically active individuals, yet their impact on health-related quality of life (HRQoL) remains insufficiently understood. This study investigated the associations between supplement use, physical activity patterns, and HRQoL in a heterogeneous sample of 537 adults engaged in sports at amateur, professional, or recreational levels. Participants completed an online survey assessing demographics, supplement use, physical activity habits, and quality of life using the SF-36 questionnaire. Statistical analyses included chi-square tests and independent-samples t-tests to explore relationships between supplement use, body mass index (BMI), motivational variables, and HRQoL outcomes. Results indicated that 46.7% of participants reported consuming at least one supplement or substance, with an average of 1.91 products. The primary motivations included performance enhancement (30.7%) and combined performance and aesthetic goals (12.1%). A significant association emerged between supplement use and the consistency of physical activity over time, as well as the individual’s motivation for engaging in exercise. Participants who maintained stable activity levels and those driven by performance or competitive motives were more likely to use supplements. In contrast, individuals exercising primarily for physical and psychological health were less likely to report supplement use. When comparing HRQoL scores, supplement users showed significantly lower levels of impairment due to emotional issues (RE), social functioning (SF), and bodily pain (BP). Among these variables, only Bodily Pain presented a small effect size, suggesting a meaningful difference between users and non-users. These findings highlight that while supplements are commonly used in athletic contexts, their association with improved quality of life is limited, and may even reflect attempts to manage physical discomfort. Further research is needed to clarify the directionality of these relationships and inform safe and evidence-based consumption.

## 1. Introduction

Participating in physical activity, regardless of whether it is at an amateur, professional, or recreational level, is generally acknowledged as fundamental to preserving and improving overall health [1,2,3]. Moreover, regular exercise provides numerous physical, psychological, and social advantages, including improved cardiovascular health, increased muscular strength, enhanced body composition, and a diminished chance of chronic diseases such as obesity, type 2 diabetes, and cardiovascular illnesses [4,5,6,7,8]. It also contributes to psychological well-being by enhancing self-esteem, improving sleep, and reducing stress and anxiety. Together, these benefits enhance Health-Related Quality of Life (HRQoL), which reflects physical, mental, and social well-being [9,10,11,12,13,14]. Moreover, enhancing performance and maximizing physical benefits has become a central goal in the context of athletic training and physical activity [15,16]. In conjunction with regular training and a balanced diet, the utilization of nutritional supplements has garnered considerable popularity among athletes and physically active individuals [17,18,19].

### 1.1. Dietary Supplement Use: Definition, Prevalence and Underlying Motivations

The International Olympic Committee defines dietary supplements as substances ingested alongside a regular diet to enhance athletic performance or obtain health advantages [20]. These supplements comprise a diverse range of compounds, including vitamins, minerals, amino acids, herbal extracts, concentrates, and metabolic products. The utilization of dietary supplements is driven by diverse objectives, including elevating energy levels, augmenting muscular growth, expediting recovery, regulating body weight, and fostering general well-being [21,22,23,24,25]. Research underscores the widespread utilization of nutritional supplements among athletes. Froiland and colleagues [26] indicated that 89% of athletes utilize dietary supplements, revealing significant differences between professional and amateur athletes. The most frequently ingested supplements are energy drinks (73%), meal replacement products (61.4%), multivitamins (47.3%), and creatine (37.2%) [27,28]. Supplement usage incentives differ according to demographic parameters, including age and gender, as well as the intensity and nature of physical activity. Specifically, professional athletes and women generally utilize nutritional supplements more [29,30,31]. More recent literature appears in line with those claims, stating that dietary supplements are widely used by athletes regardless of the sport discipline and competitive level [20]. However, the kind of supplementation employed seems to be dependent on the characteristics of the physical activity practiced and its intensity. For instance, proteins (i.e., whey, casein, branched-chain amino acids—BCAAs) are among the most used supplement categories, with consumption reaching 96% in bodybuilders, 60–77% in team sport athletes, such as rugby and American football, and 60–80% in strength and endurance sports [27,32]. Multivitamins and mineral supplements are more commonly utilized by female endurance athletes, particularly vitamin D and iron, with usage rates exceeding 50% in certain disciplines [33,34]. Creatine is recognized for its ergogenic properties and is especially favored by strength and bodybuilding athletes, with usage rates between 60% and 96% [27]. Caffeine and energy drink consumption is notably significant in team and endurance sports, with rates ranging from 30% to 50% [35,36]. Electrolytes and recovery supplements are utilized by 50–80% of endurance athletes. Omega-3 and vitamin D are notably prevalent among female soccer players and other outdoor sports athletes, with usage rates ranging from 40 to 66% [33,37]. Although nutritional supplements may improve sports performance and physical recovery, their usage has inherent risks [38,39,40]. Moreover, contamination of supplements might yield positive outcomes in doping tests, while improper or excessive consumption may result in nutritional imbalances, adverse pharmaceutical interactions, or even abnormalities in cognition and mood. These detrimental effects might adversely affect sports performance and general quality of life [41,42,43,44,45,46]. The prevalent utilization of dietary supplements prompts significant inquiries regarding their true impact on enhancing quality of life for individuals participating in sports and physical activities. Furthermore, it is uncertain if the consumption of such substances significantly improves mental health outcomes or, alternatively, whether improper use may result in adverse effects [47,48,49,50,51,52].

### 1.2. Research Gap and Aim of the Study

This study extends prior research on dietary supplement use among rugby players by broadening the scope to include a more diverse athletic population and exploring related health outcomes [53]. In the present work, the scope has been expanded to include individuals engaged in various sports, both amateur and professional levels. This broader approach allows for a more comprehensive analysis of supplement consumption patterns and their connection to HRQoL across a diverse athletic population. In fact, this cross-sectional study examines the use of dietary supplements among adults’ individuals (at least 18 years old) participating in physical activity across various levels, including amateur, professional, and recreational. This study aims to identify the motivations behind supplement intake, including performance enhancement, recovery, health maintenance, and nutritional deficiencies. Finally, this study assesses potential differences in HRQoL among supplement users and non-users, offering insights into the possible influence of supplementation and exercise on health outcomes.

## 2. Materials and Methods

### 2.1. Participants

Participants were recruited from general population and specifically through targeted outreach to athletes and sports clubs. Participants were recruited via online channels (e.g., social media, email), and the complete set of questionnaires was administered online. Participation in the survey was entirely voluntary, and no incentives or compensation were offered. The study evaluated adult participants (aged 18 years or older) engaged in competitive or amateur sports, either currently or in the past. A total of 538 athletes were selected and included in this study. Only one participant was removed due to incomplete questionnaire responses. All participants were required to read and accept an informed consent form prior to completing the questionnaire.

### 2.2. Research Design and Methodology

This non-clinical cross-sectional study was conducted from 30 July 2024 to March 2025. Participants who met the inclusion criteria were requested to provide their consent to partake in the study by consenting to the informed consent concerning data processing for scientific and research purposes, which was presented prior to their access to the questionnaires. Participants were apprised of the study’s objective and the confidentiality of the data collection and analysis. This document, together with the utilized questionnaires and the data collecting and storage methodologies, received approval from the Ethics Committee of Sigmund Freud University, the Ethics Commission of the Faculty of Psychotherapy Science, and the Faculty of Psychology. The approval reference is BD5VKJDAC4UJIC91006. To maintain privacy, no personal data that could identify subjects was collected.

### 2.3. Data Collection

The Italian version of the 36-item Short Form Health Survey (SF-36) [54] was employed to evaluate the quality of life of athletes. The SF-36 is a self-administered questionnaire designed to quantify health status and assess HRQoL. The questionnaire comprises 36 questions categorized into 8 subscales. Physical functioning (PF) (10 items) evaluates the extent of physical exercise and the impact of physical conditions on daily activities. Inquiries relate to difficulties faced in performing ordinary activities, such as walking or handling objects. Limitations due to physical health (Role Physical—RP) (4 items) assesses the degree to which physical health problems impede job and social engagements. Limitations due to emotional issues (Role Emotional—RE) (3 items) evaluates the impact of emotional elements on work performance and daily activities. Energy and dissatisfaction (Vitality—VT) (4 items) measures reported energy levels and tiredness. Psychological well-being (Mental Health—MH) (5 items) assesses mental health, including levels of anxiety and depression. Social engagement (Social Functioning—SF) (2 items) assesses the impact of physical and mental problems on social interactions and activities. Pain (Bodily Pain—BP) (2 items) evaluates the intensity and frequency of physical discomfort and its effect on daily activities, while general health perception (General Health—GH) (5 items) measures the overall assessment of health status and general well-being [54]. A low score in this assessment identifies a diminished level of well-being, whilst a high score denotes an elevated level of well-being. The overall score was calculated by computing the arithmetic mean of the SF-36 questionnaire subscales. The SF-36 has been thoroughly examined for validity and reliability in the Italian setting, demonstrating its psychometric robustness. Numerous studies indicate high test–retest reliability, with correlation coefficients exceeding 0.80 for most subscales, and satisfactory internal consistency, as evidenced by Cronbach’s α typically greater than 0.70 [55,56]. These findings validate the SF-36 as a dependable instrument for evaluating HRQoL.

A 24-item ad hoc survey was developed to collect participants’ physical exercise habits. Specifically, the following data was gathered: (1) the extent of physical activity practiced by amateur athletes; (2) the specific sport practiced; (3) the frequency and duration of athletic activity; (4) the category of dietary supplements utilized; (5) the primary motivation for supplement consumption; (6) the predominant motivation for engaging in physical activity; (7) the competitive level of physical activity; and (8) the extent of physical activity undertaken by professional athletes. To gather data on supplement usage and the motivations for their consumption, two direct multiple-choice questions were administered. To ensure that data on supplementing was correlated with participants’ engagement in physical activity, they were instructed not to refer any potential use of supplements utilized for the treatment of underlying health issues. The questionnaire also encompassed a brief description of participants’ demographic information, including gender, age, nationality, weight, and height, to provide a more accurate characterization of the surveyed population. In line with the International Olympic Committee, a broad definition of supplement use was adopted. Specifically, it was implemented a distinction between dietary supplements (e.g., energy drinks, multivitamins, whey protein, creatine, caffeine, carnitine) and other non-nutritional substances (e.g., analgesics, cannabis, laxatives, diuretics) used for performance-enhancing purposes, consistent with classifications in prior research. To ensure that data on supplementation were linked to participants’ engagement in physical activity, they were explicitly instructed not to report supplements used for the treatment of underlying health conditions. This instruction was intended to examine solely substances used in relation to physical performance, injury recovery, or general wellness.

### 2.4. Statistical Analysis

Statistical analysis began with descriptive statistics of all variables. Chi-squared tests were performed to evaluate association between dietary supplements usage, sex assigned at birth, BMI, and physical activity-related variables (i.e., physical activity regularity, prevailing motivation behind physical effort, and level of competitive involvement) with Phi (ϕ) coefficients for effect size. Differences in column proportions were assessed using z-tests, with *p*-values adjusted using the Bonferroni correction. Adjusted standardized residuals resulting from variables cross-tabulation were analyzed by converting them into chi-square values and evaluating significant deviations from expected values, also applying the Bonferroni correction. Independent-samples t-tests were subsequently employed to examine differences in HRQoL scores between dietary supplements users and non-users. Cohen’s d was used as the measure of effect size. A binary logistic regression was conducted to assess the association between HRQoL domains and supplement use. Model fit was evaluated using the Hosmer–Lemeshow test, while the significance of individual predictors was assessed using the Wald chi-squared test. In addition, a bivariate correlational analysis was performed to examine the relationship between the number of supplements consumed and HRQoL global scores. Subgroup differences in HRQoL based on physical activity-related characteristics (i.e., regularity of physical activity and competitive level of engagement) were further examined using one-way ANOVA. Normality of data distributions was evaluated using the Shapiro–Wilk test and visual inspection of Q-Q plots. The statistical significance cut-off level was set at *p* < 0.05, 2-tailed. Data were analyzed using IBM SPSS Statistics (Version 29.0.2.0).

## 3. Results

### 3.1. Descriptive Statistics

Of the total 537 athletes who participated in the survey, 326 (60.7%) were male and 211 (39.3%) were female. Most of them were Italian (98.4%), while the remaining (1.6%) reported to come from France (n = 2; 0.4%), Switzerland (n = 2; 0.4%), India (n = 1; 0.2%), Peru (n = 1; 0.2%), Russia (n = 1; 0.2%), and Slovenia (n = 1; 0.2%). The mean age of the sample was 32.44 (±13.64) years, ranging from 18 to 76 years of age. The mean BMI recorded within the sample was 24.22 (±4.07) kg/m^2^, ranging from a minimum of 15.21 kg/m^2^ to a maximum of 42.45 kg/m^2^. Participants reported engaging in regular physical activity for an average of 16.71 (±12.08) years, spanning from less than a year to 60 years. Detailed information on the physical activity-related characteristics reported by the sample is outlined in Table 1.

Regarding supplements and/or other substance usage, 46.7% of the participants (n = 251) reported that they consumed at least one supplement and/or other substance during their physical activities, whereas the remaining 53.3% did not (n = 286). The average number of supplements and/or other substances used by the athletes was 1.91 (±1.54), ranging from a minimum of one to a maximum of eight dietary products currently taken. Frequencies concerning the prevailing motivation behind supplements and/or other substance consumption as well as the specific type of supplements used are provided in Table 2.

### 3.2. Supplements Usage, Sex, Body Mass Index, and Physical Activity

Chi-square tests of independence were conducted to examine the associations be-tween dietary supplements use and sex, BMI, as well as between dietary supplements use and physical activity-related variables. A significant association was obtained between sex assigned at birth and supplements consumption (χ^2^(1) = 12.08, *p* < 0.001, ϕ = 0.150). Ex-amination of the adjusted standardized residuals showed that males were significantly more likely to use dietary supplements than expected (z-score = 3.50, *p*-adjusted < 0.001), and significantly less likely to not use them (z-score = −3.50, *p*-adjusted < 0.001). On the other hand, females were underrepresented among dietary supplements users (z-score = −3.50, *p*-adjusted < 0.001) and overrepresented among non-users (z-score = 3.50, *p*-adjusted < 0.001). No association was observed between BMI and dietary supplements use in our sample (χ^2^(3) = 3.17, *p* = 0.367, ϕ = 0.077). However, a significant association was detected between supplements use and physical activity regularity (χ^2^(2) = 17.23, *p* < 0.001, ϕ = 0.179; see Table 3). Examination of the adjusted standardized residuals showed that participants who reported having been more physically active in the past, compared to present, were significantly less likely to use dietary supplements than expected (z-score = −3.90, *p*-adjusted < 0.001), and significantly more likely to not use them (z-score = 3.90, *p*-adjusted < 0.001). Conversely, athletes who reported that their engagement in physical activity remained relatively stable over the years were overrepresented among dietary supplements users (z-score = 4.02, *p*-adjusted < 0.001) and underrepresented among non-users (z-score = −4.02, *p*-adjusted < 0.001). No other cells showed statistically significant deviations from the expected frequencies.

Furthermore, a significant association was obtained between dietary supplements use and the primary motivation for engaging in physical activity (χ^2^(4) = 29.50, *p* < 0.001, ϕ = 0.234; see Table 4). Examination of the adjusted standardized residuals indicated that individuals who reported engaging in physical activity to enhance their athletic performance were significantly more prone to using dietary supplements than expected (z-score = 3.92, *p*-adjusted < 0.001), and significantly less prone to not use them (z-scores = −3.92, *p*-adjusted < 0.001). Likewise, subjects who engage in physical activity for competitive purposes were overrepresented among supplements users (z-scores = 2.49, *p*-adjusted < 0.05) and underrepresented among supplement non-users (z-scores = −2.49, *p*-adjusted < 0.05). Yet, participants who engage in physical activity to enhance their physical and psychological health were significantly less likely to use dietary supplements than expected (z-score = −3.39, *p*-adjusted < 0.01), and significantly more prone to not use them (z-scores = 3.39, *p*-adjusted < 0.01). No other cells showed statistically significant deviations from expected frequencies. No association was observed between supplements use and agonistic-level physical activity (χ^2^(2) = 1.09, *p* = 0.579, ϕ = 0.045).

### 3.3. Supplements Usage and Health-Related Quality of Life

Independent samples t-tests were conducted to compare the general HRQoL scores collected from subjects who consume supplements and/or other substances with those collected from subjects who do not use them. A trend toward a statistically significant difference in SF-36 global scores was observed between the two groups (Group (Non-users): 73.39 ± 13.39; Group (Users): 71.02 ± 14.27; *t*(535) = 1.95, *p* = 0.051, *d* = 0.17). Moreover, statistically significant differences in SF-36 Role Emotional (Group (Non-users): 73.19 ± 37.41; Group (Users): 66.27 ± 39.04; *t*(535) = 2.09, *p* < 0.05, *d* = 0.18), Social Functioning (Group (Non-users): 66.30 ± 24.56; Group (Users): 62.10 ± 24.64; *t*(535) = 1.97, *p* < 0.05, *d* = 0.17), and Bodily Pain (Group (Non-users): 84.34 ± 19.13; Group (Users): 79.86 ± 20.59; *t*(535) = 2.62, *p* < 0.01, *d* = 0.23) subscales were observed between the two groups, with users of supplements and/or other substances reporting worse HRQoL scores in all three domains compared to non-users (see Table 5).

Furthermore, a binary logistic regression was computed to assess the relationship be-tween HRQoL and supplements and/or other substance consumption (Cox & Snell R2 = 0.042; Hosmer and Lemeshow test: χ^2^(8) = 10.329, *p* = 0.243). Among HRQoL domains, physical functioning (χ^2^(1) = 4.046, *p* = 0.044), bodily pain (χ^2^(1) = 7.998, *p* = 0.005), and general health (χ^2^(1) = 4.658, *p* = 0.031) were significant unique predicators of supplements usage. A bivariate correlation was also calculated to test the relationship between the number of supplements and/or other substances used and athletes’ HRQoL levels, indicating that a lower consumption of supplements was associated with higher HRQoL global scores (r(535): −0.111, *p* = 0.010).

### 3.4. Sex, Physical Activity, and Health-Related Quality of Life

Additional Independent samples *t*-tests were conducted to examine differences in HRQoL based on sex assigned at birth, revealing that males reported significantly higher global scores than females (Group (Males): 73.64 ± 13.62; Group (Females): 70.21 ± 13.96; *t*(535) = 2.82, *p* < 0.01, *d* = 0.25). Moreover, one-way ANOVA revealed significant differences in HRQoL levels based on the regularity of physical activity (F(2,534) = 5.048, *p* = 0.007, η^2^ = 0.02). Specifically, post hoc tests with Tukey correction revealed a significant difference in HRQoL global scores between athletes who maintained relatively consistent physical activity over the years and those who reported engaging in less physical activity in the past compared to the present (Group (constant physical activity over the years): 73.53 ± 13.69; Group (less physical activity than now): 67.18 ± 14.96; *p* = 0.011). No significant differences were observed in HRQoL global scores based on agonistic-level physical activity (F(2,534) = 0.613, *p* = 0.542).

## 4. Discussion

In this study, nearly half of the participants (46.7%) reported using at least one supplement or substance in the context of their physical activity. This proportion indicates a widespread reliance on supplementation as a perceived support for physical or performance-related outcomes. On average, users reported consuming approximately two products, with some individuals reporting the use of up to eight different substances. This variation likely reflects differences in training goals, intensity, and nutritional awareness [57,58,59]. Moreover, self-reported motivations for supplement use centered primarily on performance enhancement (30.7%), followed by body appearance-related reasons, either alone (3.9%) or in combination with performance (12.1%). These findings highlight that, for a significant portion of users, supplementation is not merely an adjunct to training, but rather a strategic component of broader self-optimization efforts including both aesthetic and functional objectives [60,61,62,63,64,65,66]. Comparing the data obtained in this study with our prior research [53], which concentrated solely on rugby athletes, the current survey revealed a greater prevalence of supplement users (57.6% compared to 46.7%), and greater disparities in HRQoL between users and non-users, especially in the domains of mental health, emotional functioning, and social functioning. However, it is important to note that the effect sizes in the rugby-specific sample were moderate, whereas the present, broader sample of athletes generally exhibited negligible differences. This contrast may be indicative of the distinctive physical and psychological requirements of rugby, a contact sport that is characterized by a high risk of injury, intense physical training, and performance pressure [67]. These factors may affect both the motivation to use supplements and their potential psychosocial effects. These disparities highlight the necessity of sport-specific evaluations when assessing supplementing practices and their correlation with reported well-being.

Furthermore, the types of supplements and substances reported in the current study reflect a usage profile aligned with current trends in the literature. Frequently used products included multivitamins and whey protein, indicating a preference for widely available and generally accepted supplements [68,69,70,71]. The presence of more targeted ergogenic aids (i.e., nutritional supplements that can enhance performance efficiency such as creatine and caffeine) also reflect performance-driven intentions. Notably, the reported use of substances not typically categorized as sports supplements (i.e., analgesics, cannabis, diuretics, and laxatives) raises concerns about unsupervised or potentially inappropriate strategies aimed at influencing performance, body composition, or recovery [38,72,73,74,75,76].

### 4.1. Physical Activity, Supplementation and BMI

Moreover, this study revealed no statistically significant association between Body Mass Index (BMI) and dietary supplement use among physically active individuals. This result suggests that supplement consumption in this population is not directly linked to general body composition and may instead be influenced by other factors such as training habits, perceived needs, or individual motivations. This result aligns with prior evidence indicating that BMI alone may be a limited predictor of supplement use, especially within athletic or active samples, where weight does not necessarily reflect health behaviors or performance goals [77,78,79,80]. In contrast, a significant association emerged between supplement use and the perceived regularity of physical activity over time. Specifically, participants who maintained consistent levels of physical activity throughout the years were more likely to report using dietary supplements. On the other hand, those who reported a reduction in activity compared to the past were significantly less likely to use supplements [81,82,83,84]. This pattern may suggest that consistent engagement in physical activity fosters greater investment in supporting behaviors such as supplementation [27,85,86]. Moreover, supplement use was strongly associated with individuals’ primary motivation for engaging in physical activity. Participants who exercised to enhance athletic performance or for competitive reasons were significantly more likely to use supplements [87,88,89]. These findings support the idea that supplement use is not merely a reflection of training volume or athletic status per se, but rather of goal orientation.

### 4.2. Potential Risks

These findings suggest that some individuals may select supplements without professional advice, relying instead on informal sources such as peers or social media [90]. The inclusion of pharmacologically active substances further underscores the need for improved education around supplement safety, efficacy, and ethical considerations, especially among non-professional or recreational athletes who may be more vulnerable to misinformation [91,92,93]. Performance-driven motivations may prompt individuals to seek ergogenic or recovery-supportive aids, regardless of whether they compete at an agonistic level [94,95,96,97]. Interestingly, no association was found between supplement use and current or past engagement in competitive-level sports, suggesting that perceived goals may carry more weight than formal competition status in shaping supplementation behavior. Conversely, participants who identified health and well-being as their primary motivation for engaging in physical activity were significantly less likely to use dietary supplements [98,99,100]. This finding may reflect a preference for naturalistic or holistic approaches among individuals oriented toward general wellness, who might perceive supplementation as unnecessary. It may also indicate different levels of exposure to performance culture, or divergent sources of information regarding supplementation [101,102,103]. Altogether, these results underscore the importance of psychological and motivational factors in predicting supplement use, possibly more so than demographic or anthropometric variables. Understanding the underlying drivers of supplementation behaviors can inform the development of targeted educational interventions, particularly those aimed at promoting safe and evidence-based use of supplements within diverse physically active populations [104,105]. The comparison between users and non-users of dietary supplements and/or other substances revealed statistically significant differences in several dimensions of HRQoL, as assessed by the SF-36 questionnaire [106,107]. Although a trend toward significance was observed for the overall SF-36 global score, the effect size was small (d = 0.17), indicating only a minimal difference between the two groups in perceived general well-being.

More specifically, significant differences were found in the Role Emotional, Social Functioning, and Bodily Pain subscales, with supplement users reporting lower scores in all three domains. However, it is important to interpret these findings considering the effect sizes. According to Cohen’s classification, the differences observed in the Role Emotional (d = 0.18) and Social Functioning (d = 0.17) subscales are negligible to very small in practical terms. Only the difference found in the Bodily Pain subscale reached the threshold for a small effect size (d = 0.23), suggesting that users of supplements and/or substances may experience slightly more physical discomfort or pain than non-users [108,109,110,111]. This finding is particularly relevant given that bodily pain is a physical dimension that may directly impact training routines and recovery. One possible interpretation is that individuals experiencing higher levels of bodily discomfort may turn to supplements to alleviate symptoms or improve recovery outcomes. Alternatively, increased use of certain substances could itself be associated with adverse effects, either directly (i.e., using stimulating or dehydrating agents) or indirectly (i.e., through overtraining encouraged by perceived support from supplements) [112,113,114,115,116,117].

### 4.3. HRQoL and Supplements Use

Moreover, the lack of moderate or large effect sizes in the SF-36 areas suggests that, in general, using supplements does not have a strong link to better or worse HRQoL for this group. However, the trend showing that supplement users often report slightly worse health outcomes might indicate that there are other health issues, injury rates, or mental health factors involved that are not just about supplements consumption. To further explore the relationship between health-related quality of life and supplement use, a binary logistic regression analysis was performed. This model identified physical functioning, bodily pain, and general health as significant independent predictors of supplement and/or other substance use [118,119]. These findings suggest that perceived physical discomfort and overall health status may play a meaningful role in shaping individuals’ decisions to engage in supplementation [120,121,122,123]. Importantly, these predictors offer a possible understanding of the motivational and health-related dimensions that may underlie supplement use, extending the insights gained from the group comparisons. An alternative explanation for the observed lower HRQoL scores among supplement users may relate to individual characteristics not directly measured in the present study. For example, previous research suggests that supplement use is sometimes more frequent among individuals who are more health-conscious, but also those with elevated health-related anxiety or subclinical hypochondriacal tendencies. Such individuals may report lower subjective well-being, regardless of their actual health status, and may turn to supplementation as a perceived form of self-care or control [124,125,126]. Socioeconomic and educational variables, although not assessed in this study, may also play a role in shaping both supplement use and perceptions of well-being. For example, individuals with higher socioeconomic status may have greater access to commercial supplements and health-related information, leading to more frequent or varied usage patterns [127]. Conversely, those with lower levels of education might rely more heavily on informal sources of information (e.g., social media, peers), potentially resulting in less evidence-based or riskier supplement behaviors [21,128]. These factors could therefore mediate or moderate the relationship between supplement use and reported HRQoL, by shaping both actual behaviors and subjective perceptions. Future research should include such variables to better contextualize the psychological and behavioral patterns observed in supplement users. Subgroup analyses showed that males reported significantly higher HRQoL scores compared to females, and participants who maintained consistent physical activity over the years exhibited better HRQoL than those whose activity levels had recently increased. Additionally, no significant differences were found based on competitive level. These results suggest that gender and long-term engagement in physical activity may be associated with perceived quality of life, independently of formal athletic status [128,129]. Moreover, further studies are needed to explore the causal direction of these associations and to identify mediating variables that may explain why individuals with more bodily pain or emotional burden are more likely to engage in supplementation behaviors.

## 5. Limitations and Further Directions

This study presents several limitations. First, its cross-sectional design does not allow for causal inferences between dietary supplement use, physical activity, and HRQoL. Second, data were collected via self-reported questionnaires, which may introduce biases such as memory errors, social desirability, and subjective interpretation of questions [130]. Third, although the sample included a broad range of sports disciplines and competitive levels, most participants were Italian, potentially limiting the generalizability of findings to other cultural or national contexts.

### 5.1. Unaddressed Variables and Instrument Limitations

This study did not account for important characteristics of supplement use such as type, dosage, duration, or medical supervision, all of which may influence their effects on HRQoL. Moreover, while the SF-36 is a well-validated instrument, it provides a general measure of quality of life and may not fully capture sport-specific dimensions relevant to athletes. Motivational factors, such as perfectionism, body image dissatisfaction, perceived competence, and self-determination in sport, could further explain differences between users and non-users [131,132], acting as mediators or moderators in the relationship between supplementation and well-being. Individual psychological traits, e.g., health-related anxiety or subclinical hypochondriasis, may also play a role, influencing both supplement behaviors and subjective well-being. Additionally, socioeconomic and educational factors could affect access to supplements and health information, shaping usage patterns and health perceptions. Including these variables in future research would allow for a more accurate contextualization of the observed associations. The high heterogeneity of the sample, in terms of athletic level (amateur, recreational, professional), gender distribution, and motivations for both physical activity and supplement use, may have introduced substantial variability, potentially diluting specific associations or moderating effects [131,132]. Although the overall sample size was relatively large, some subgroups (e.g., performance-oriented athletes or users of specific supplements) were underrepresented, limiting the statistical power for detailed analyses [20,133,134,135,136,137]. For this reason, we did not perform comparisons across athletic levels (e.g., professional vs. recreational athletes), as the resulting inferences would likely lack robustness and generalizability. Future studies with more balanced subsamples are needed to explore these potential group-specific differences.

### 5.2. Future Research

Future studies should broaden the sample to include diverse populations across countries, sports disciplines, age groups, and competition levels, thus enhancing generalizability and enabling sport-specific analyses. Longitudinal designs would be especially valuable to explore causal relationships, monitor supplementation over time, and assess how changes in physical activity, health conditions, or psychological well-being affect supplement behavior. Qualitative approaches, e.g., interviews or open-ended questionnaires, could provide deeper insights into individual motivations for supplement use, including societal, emotional, or performance-related drivers. Research should also incorporate detailed data on supplement type, dosage (e.g., milligrams), frequency, duration, and medical supervision. Furthermore, collecting background variables, such as socioeconomic status, education level, and pre-existing medical conditions, would allow for better control of potential confounders. Finally, integrating biological or performance-related indicators, e.g., injury rates, recovery markers, training load, with self-report measures could improve understanding of how supplements affect health and athletic performance.

## 6. Conclusions

This study explored the relationship between dietary supplement use, physical activity, and HRQoL among athletes and physically active individuals. The findings revealed that nearly half of the participants reported using supplements or substances, primarily for performance enhancement or aesthetic purposes. While supplement use was not associated with BMI or competitive level, it was related to the regularity of physical activity and motivation behind exercise. Moreover, users reported slightly lower HRQoL scores than non-users, with the only small—but meaningful—difference observed in the Bodily Pain subscale. These results suggest that supplementation practices may not necessarily translate into perceived improvements in quality of life and, in some cases, could be associated with physical discomfort or overtraining behaviors. Understanding the motivational and behavioral profiles of supplement users could inform targeted educational strategies promoting safe and evidence-based practices in physically active population.

## Figures and Tables

**Table 1:** Summary of the physical activity-related characteristics of the sample.

Variable	Frequency	Percent	Cumulative Percent
Physical activity regularity			
I used to practice less physical activity than I do now	45	8.3%	8.3%
I used to practice more physical activity than I do now	155	28.9%	37.2%
My physical activity has been generally constant over the years	337	62.8%	100%
Prevailing motivation behind physical activity			
Improvement of athletic performance	44	8.2%	8.2%
Competition	45	8.4%	16.6%
Entertainment and social occasions	53	9.9%	26.5%
Improvement of body image	53	9.9%	36.4%
Improvement of physical and psychological health	342	63.6%	100%
Agonistic level physical activity			
I’ve never competed at the agonistic level	87	16.2%	16.2%
I currently compete at the agonistic level	210	39.1%	55.3%
Not currently, but I have competed at the agonistic level in the past	240	44.7%	100%
Professional level physical activity			
I currently compete at the professional level	4	0.7%	0.7%
Not currently, but I have competed at the professional level in the past	37	6.9%	7.6%
I’ve never competed at the agonistic level	496	92.4%	100%

**Table 2:** Summary of the supplement usage-related characteristics of the sample.

Variable	Frequency	Percent	Cumulative Percent
Prevailing motivation behind supplements usage			
Improvement of body image	21	8.4%	8.4%
Improvement of athletic performance	165	65.7%	74.1%
Improvement of body image and athletic performance	65	25.9%	100%
Type of supplements used			
Multivitamins or minerals	210	39.1%	-
Whey proteins	146	27.2%	-
Energy drinks	106	19.7%	-
Creatine	104	19.4%	-
Caffeine	86	16.0%	-
Carnitine	48	8.9%	-
Analgesics	46	8.6%	-
Cannabis	16	3.0%	-
Diuretics	6	1.1%	-
Laxatives	3	0.6%	-

**Table 3:** Summary of the observed and expected frequencies resulting from the cross-tabulation of physical activity regularity and dietary supplements use.

Variable	Non-Users	Users	Total
I used to practice less physical activity than I do now	26 ^a^ (23.97)	19 ^a^ (21.03)	45 (45)
I used to practice more physical activity than I do now	103 ^a^ (82.55)	52 ^b^ (72.45)	155 (155)
My physical activity has been generally constant over the years	157 ^a^ (179.48)	180 ^b^ (157.52)	337 (337)
Total	286 (286)	251 (251)	537 (537)

Note. Observed frequencies are presented outside of brackets, while expected frequencies are enclosed within brackets. Subscript letters indicate subsets of supplement use categories with column proportions that are not significantly different from one another at the 0.05 significance level.

**Table 4:** Summary of the observed and expected frequencies resulting from the cross-tabulation of prevailing motivation for engaging in physical activity and supplements use.

Variable	Non-Users	Users	Total
Competition	16 ^a^ (23.97)	29 ^b^ (21.03)	45 (45)
Entertainment and social occasions	35 ^a^ (28.23)	18 ^b^ (24.77)	53 (53)
Improvement of body appearance	23 ^a^ (28.23)	30 ^a^ (24.77)	53 (53)
Improvement of physical and psychological health	201 ^a^ (182.15)	141 ^b^ (159.85)	342 (342)
Enhancement of athletic performance	11 ^a^ (23.43)	33 ^b^ (20.57)	44 (44)
Total	286 (286)	251 (251)	537 (537)

Note. Observed frequencies are presented outside of brackets, while expected frequencies are enclosed within brackets. Subscript letters indicate subsets of supplement use categories with column proportions that are not significantly different from one another at the 0.05 significance level.

**Table 5:** Summary of the independent-samples *t*-tests.

Variable	Users (Mean ± Standard Deviation)	Non-Users (Mean ± Standard Deviation)	*t*-Value	*p*-Value	Cohen’s *d*
Global Score	71.02 ± 14.27	73.39 ± 13.39	1.95	0.051	0.169
General Health	67.51 ± 14.64	65.44 ± 15.36	1.60	0.111	0.138
Mental Health	63.38 ± 16.60	64.36 ± 16.70	0.68	0.494	0.059
Role Physical	78.78 ± 31.16	83.30 ± 28.31	1.76	0.079	0.152
Role Emotional	66.27 ± 39.04	73.19 ± 37.41	2.09	0.036	0.181
Physical Functioning	95.12 ± 8.99	94.06 ± 11.54	1.18	0.239	0.102
Social Functioning	62.10 ± 24.64	66.30 ± 24.56	1.97	0.049	0.171
Bodily Pain	79.86 ± 20.59	84.34 ± 19.13	2.62	0.009	0.226
Vitality	55.40 ± 15.61	56.08 ± 15.42	0.51	0.610	0.044

## Data Availability

The data that support the findings of this study are available from the corresponding author upon reasonable request.
